# Single Molecule Microscopy Reveals an Increased Hyaluronan Diffusion Rate in Synovial Fluid from Knees Affected by Osteoarthritis

**DOI:** 10.1038/srep21616

**Published:** 2016-02-12

**Authors:** Hendrik Kohlhof, Sascha Gravius, Sandro Kohl, Sufian S. Ahmad, Thomas Randau, Jan Schmolders, Yorck Rommelspacher, Max Friedrich, Tim P. Kaminski

**Affiliations:** 1 Department of Orthopedic Surgery and Traumatology, University Hospital und University of Bonn, Germany; 2 Department of Orthopedic Surgery and Traumatology, University Hospital und University of Bern, Switzerland; 3 Institute of Physical and Theoretical Chemistry, Rheinische Friedrich-Wilhelms Universität, Bonn, Germany

## Abstract

Osteoarthritis is a common and progressive joint disorder. Despite its widespread, in clinical practice only late phases of osteoarthritis that are characterized by severe joint damage are routinely detected. Since osteoarthritis cannot be cured but relatively well managed, an early diagnosis and thereby early onset of disease management would lower the burden of osteoarthritis. Here we evaluated if biophysical parameters of small synovial fluid samples extracted by single molecule microscopy can be linked to joint damage. In healthy synovial fluid (ICRS-score < 1) hyaluronan showed a slower diffusion (2.2 μm^2^/s, N = 5) than in samples from patients with joint damage (ICRS-score > 2) (4.5 μm^2^/s, N = 16). More strikingly, the diffusion coefficient of hyaluronan in healthy synovial fluid was on average 30% slower than expected by sample viscosity. This effect was diminished or missing in samples from patients with joint damage. Since single molecule microscopy needs only microliters of synovial fluid to extract the viscosity and the specific diffusion coefficient of hyaluronan this method could be of use as diagnostic tool for osteoarthritis.

Osteoarthritis is the most common joint disorder and a major cause of disability^1^. It is estimated that 10–15% of the worldwide population over 60 has some degree of osteoarthritis^2^.

Osteoarthritis is a progressive multifactorial disease, marked by a breakdown of the articular cartilage by heterogeneous causes including abnormal metabolic processes, mechanical injury, joint instability, excessive overweight, peripheral neuropathy and ageing^3^. Lubrication of healthy joints is provided by the interplay of articular cartilage at the bone ends in combination with the synovial fluid in between^4^. This composition provides remarkably low friction and low wear^5^,^6^. The breakdown of the lubricating properties leads to articular cartilage degradation, osteophyte formation, subchondral sclerosism, meniscal degeneration, bone marrow lesions and synovial proliferation^3^,^7^. Patients affected by osteoarthritis suffer from pain, limitations of movement and ultimately loss of joint function. Currently, there is no cure for osteoarthritis available, except total knee replacement, which still leads to loss of joint function^8^. The symptoms of osteoarthritis can still be managed and progression usually can be slowed down through patient education, weight loss, muscle training and hyaluronan injections^9^.

An obstacle to disease management strategies is the diagnosis of osteoarthritis^7^,^10^. Currently osteoarthritis diagnosis uses radiographic criteria and clinical symptoms^11^. However, by that only late stages of osteoarthritis are recognized. Therapies that aim to slow down disease progression would benefit from early diagnosis that enables an early onset of disease management.

It is projected that in 2050, 130 million people worldwide will suffer from osteoarthritis and 40 million people will be disabled by osteoarthritis^12^. This illustrates the increasing burden of osteoarthritis on communities, healthcare systems and the economy.

Next to finding a cure, strong efforts are made to find biomarkers that allow early diagnosis of osteoarthritis and cost effective long-term monitoring of patients. Imaging techniques like quantitative MRI are able to provide better diagnosis of early osteoarthritis, but costs and availability are limiting its widespread use^7^. Therefore, there is currently an unmet need for biochemical or biophysical markers that can be measured in blood, urine or synovial fluid samples^11^.

It is known that the overall viscosity of synovial fluid decreases in osteoarthritis. Therefore, the viscosity of synovial fluid was thought to be a potential biomarker for osteoarthritis. However, the viscosity of synovial fluid shows in general a large variance; therefore, its values can be misleading^13^.

Analyzing the rheological properties at a microscopic scale showed that HA forms a dynamic network in synovial fluid^14^. The existence of a dynamic network in synovial fluid is also supported by studies showing that interplay between the major macromolecular molecules of synovial fluid hyaluronan, type II collagen fibrils and PRG4 is needed for efficient lubrication^15^,^16^. In a previous study, also using single molecule microscopy, a significant reduction of HA diffusion speed could be detected in synovial fluid from a patient without a prior history of osteoarthritis. In contrast, in synovial fluid from a patient with advanced osteoarthritis there was no reduction of HA diffusion speed^17^. These results suggest a loss of hyaluronan interactions in synovial fluid during osteoarthritis that can be detected via single molecule microscopy.

Single molecule microscopy is a very powerful technique to probe the properties of a single molecular entity such as hyaluronan in complex biological environments using selective labeling. Single molecule microscopy provides a high spatial and temporal resolution. This method allows extracting even coarse structural information of single molecules. By using this method, it could be confirmed that hyaluronan molecules form random coil structures in buffer solution^17^,^18^.

Here we measured the diffusion coefficient of fluorescent hyaluronan (fl-HA) and compared it to the diffusion of fluorescent dextran (fl-dextran), an inert tracer, in human synovial fluid. We analyzed synovial fluid samples of patients without joint damage (w/o-JD) and with joint damage (w/-JD).

The diffusion coefficient of spherical objects depends on its hydrodynamic radius, the viscosity of the surrounding medium and temperature. The Stokes-Einstein equation describes the dependency of diffusion coefficient and these three variables (see M&M) eq. 2). Higher viscosities or larger hydrodynamic radii result in a lower diffusion coefficient. Higher temperatures result in a higher diffusion coefficient.

The diffusion coefficient of fl-dextran corresponds to the viscosity of the sample^17^. For hyaluronan it is known that it can undergoes interactions in synovial fluid. The hydrodynamics radius of such conjugates would be larger and therefore its diffusion coefficient smaller.

Single molecule microscopy observation of tracer molecules in human synovial fluid samples, as conducted here (Fig. 1), would be feasible with standard microscopy equipment. With minimal (microliter) amounts of synovial fluid accurate quantitative measurements of diffusion coefficients is possible.

## Results

### Viscosity of synovial fluid shows large variability

First, the diffusion coefficient of fl-Dextran (D_dex_) and fl-HA (D_HA_) was determined in buffer solution (Supplementary Fig. S1). Due to its lower molecular weight fl-HA has a higher diffusion coefficient as fl-Dextran in buffer solution.

**Figure 2:**
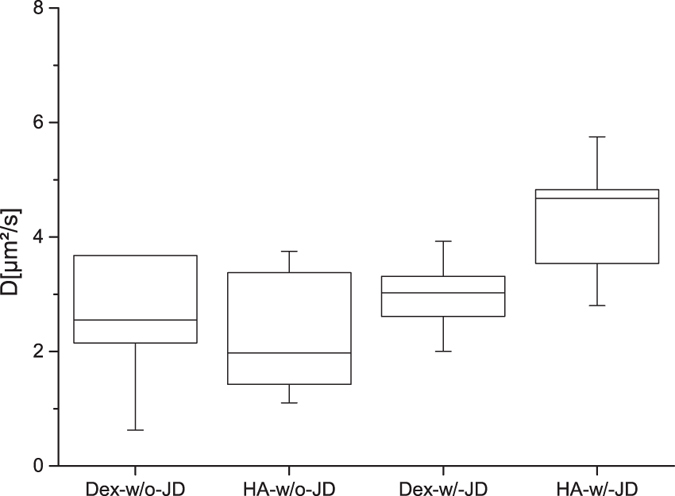
Hyaluronan diffusion is slowed down in synovial fluid from patients without joint damage but not in joints affected by osteoarthritis. The viscosity of synovial fluid sample of patients affected by joint damage (w/-JD) tends to be lower, as indicated by the on average higher diffusion coefficient of fl-dextran. The overlap between both patient cohorts makes it unfeasible to diagnose joint damage based on synovial fluid-viscosity. Whereas, the diffusion coefficient of fl-HA is significantly reduced in the w/o-JD-group compared to samples collected from patients affected by joint damage. Nevertheless, the HA diffusion coefficients still show an overlap between patients with and without joint damage.

Fl-Dextran is a commonly used inert probe in single molecule microscopy and its diffusion coefficient (D_Dex_) corresponds to the viscosity of the sample. D_Dex_ scatter over a wide range for both patient cohorts (Fig. 2). D_Dex_ of the w/o-JD-group and w/-JD -group show a large overlap and do not differ significantly. Because of their large overlap, it is impossible to distinguish the w/-JD -group from the w/o-JD-group based only on D_Dex_ respectively synovial fluid viscosity.

Three patients showed joint damage (ICRS > 2) during knee arthroscopy. The samples of these patients were assigned to the w/-JD-group. Interestingly, D_HA_ and D_dex_ of these three patients (Fig 2. encircled data points) did not differ from the other subjects in the w/-JD-group, which were in need of total knee arthrosplasty.

### Slowed hyaluronan diffusion is a characteristic feature of healthy synovial fluid

In contrast to D_Dex_, D_HA_ between patients with and without joint damage differs. The diffusion of fl-HA is significantly slower in the w/o-JD-group (2.2 μm^2^/s) compared to the w/-JD-group (4.6 μm^2^/s) (P = 0.01) (Fig. 2).

While average D_HA_ is slower in the w/-JD-group compared to the w/o-JD-group, the distributions of D_HA_ still show a great overlap. Therefore, D_HA_ alone is no suitable marker to detect joint damage, either.

**Figure 3:**
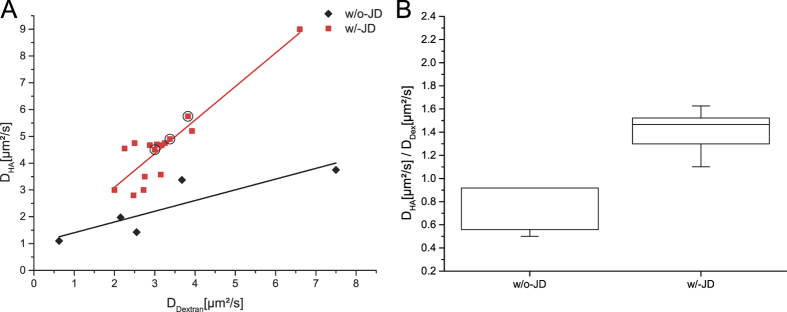
(**A**) Linear regression of D_HA_ vs. D_Dex_ shows a selective reduction of HA diffusion coefficient in synovial fluid from patients without joint damage (slope = 0.4 +/− 0.1). This reduction is missing synovial fluid from patients affected by joint damage (slope 1.3 +/− 0.2). The slope of the w/-JD-group resembles the ratio of D_HA_ and D_Dex_ found in buffer solution and indicates that in patients with osteoarthritis, HA undergoes no or less interactions. The encircled data points represent patients not in need of total knee arthroplasty but with an ICRS-score > 2. (**B**) Analyzing D_HA_/D_Dex_ makes patients with joint damage distinguishable from patients without joint damage.

While D_HA_ and D_Dex_ of both patient cohorts overlap at least to some extent, the w/o-JD-group becomes distinguishable from the w/-JD-group by correlating D_HA_ and D_Dex_ (Fig. 3).

By just increasing the overall viscosity of the fluid, the ratio of the diffusion coefficients of fl-HA and fl-Dextran stay the same (see eq. 2).

D_HA_ and D_Dex_ show a linear relationship with a high correlation coefficient in the w/o-JD- and w/-JD-group (0.88 & 0.9). The slope of the linear regression in the w/-JD-group (1.3 +/− 0.2) is concurrent with the ratio of D_HA_ vs. D_Dex_ in buffer (1.3 +/− 0.1) (Fig. 3). In contrast to this, the slope of D_HA_ vs. D_Dex_ is reduced to 0.4 +/− 0.1 in the w/o-JD group.

## Discussion

The ratio of D_HA_ and D_Dex_ found in our study allows the distinction of patients with and without joint damage. Furthermore, patients with advanced osteoarthritis had a similar D_HA_/D_Dex_ ratio as patients who needed total knee arthrosplasty. Therefore, we suggest the D_HA_/D_Dex_ ratio could be a suitable marker for osteoarthritis.

A decreased synovial fluid viscosity was linked to pathologic joint conditions of patients suffering from osteoarthritis in several studies. Suitability of synovial fluid viscosity as an indicator for osteoarthritis or joint damage has been discussed controversial^13^,^19^. Its viscosity is influenced by several parameters such as age and physical activity. Several studies show a large variance of synovial fluid viscosity between different individuals^19^,^20^,^21^. Our results show a large overlap of synovial fluid viscosity between samples of patients with and without joint damage. The large overlap shows that synovial fluid viscosity alone is not a reliable marker for osteoarthritis.

More strikingly, we found that in synovial fluid samples fl-HA showed a slower diffusion than expected from the sample viscosity. In contrast to that, in samples from patients with joint damage this effect was diminished or missing. The diffusion coefficient of fl-HA in samples from patients with joint damage corresponded well to the sample viscosity.

As previously described, the well-balanced interaction of the main macromolecular synovial fluid components HA, PRG4 and collagen type II fibrils are important for the low coefficient of friction in healthy joints^15^. Progression of osteoarthritis is linked to a higher coefficient of friction. In line with that results our data show that HA undergoes intermolecular interactions in healthy joints and these interactions are lost in pathological changed joints. The ratio of D_HA_/D_Dex_ presumably allows the discrimination of patients affected by osteoarthritis from patients with no hyaline damage. Therefore, we suggest that the D_HA_/D_Dex_ ratio could be used to monitor pathological changes in this interplay and thereby the detection of early osteoarthritis.

Furthermore, our data raise the question of whether loss of intermolecular interactions of hyaluronan in synovial fluid is cause or consequence of osteoarthritis. This will be addressed in future studies.

Single molecule microscopy requires only small sample volumes (10–100 μl). Furthermore, to its extreme sensitivity only picomolar amounts of tracer molecules are needed. Such low concentrations are not changing the sample properties and reduce the required amount of tracer molecules dramatically. The samples need no further preparation before measurement. The experimental procedure and data analysis can be automated. By using state-of-the-art automation equipment, several hundred samples could be analyzed per microscope per day. Furthermore, the required image analysis algorithms are already freely available helping to keep the costs of this method low.

Thus, single molecule microscopy is a useful method for probing the organization of human synovial fluid and shows potential for diagnosis of early osteoarthritis.

## Material and Methods

### Single Molecule Microscopy

Single-particle imaging experiments were performed using a custom-built single molecule microscope based on a Zeiss Axiovert 200 TV equipped with a 100 × NA 1.4 oil immersion objective lens (Carl Zeiss Microimaging GmbH, Jena, Germany). Fluorescence was excited at 532 nm by a diode-pumped solid-state laser (Pluto Pegasus Lasersysteme GmbH, Wallenhorst, Germany). Selective plane illumination was achieved via HiLo-illumination. Laser illumination was switched on only during image acquisition by means of an acousto-optical tunable filter (AA Optoelectronics, Orsay Cedex, France). For single-particle image acquisition, we used the iXon DV-860 BI camera (Andor Technology, Belfast, Northern Ireland) in combination with a 4× magnifier and 3x binning yielding a pixel size in the object space of 120 nm. Generally, 2500 frames were recorded in a single movie, with a frame rate of *k*_acq_ = 200 Hz. All measurements were performed at 22 °C. Since synovial joints are avascular compartments, many of which are located in the periphery of the body, it seems reasonable to assume that the temperature in peripheral joints exposed to the ambient can be substantially lower than 37 °C. It is assumed that the synovial fluid components can still provide their lubricating properties at 22 °C in a physiological relevant manner. The temperature of 22 °C was meant to approximate the conditions in small peripheral joints.

### Image analysis

Identification of the single-molecule signals and tracking was done with Matlab using a self-written single particle-tracking algorithm. Analysis of the trajectory data as described before was done with a in house built Matlab program^17^.

### Mathematical analysis of the jump distance distribution

The jump distance distribution of a molecule with diffusion coefficient *D* is given by


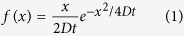


While the diffusion coefficient D of spherical objects is described by the Stokes-Einstein equation


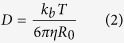


Whereas, *k*_*b*_ denotes the Boltzmann constant, *T* absolute temperature, *η* the viscosity and *R*_*0*_ the hydrodynamic radius.

### Statistical Testing

For calculation of all P values, two-tailed Mann-Whitney test was used.

### Preparation of fluorescent probes

Fluorescent HA (fl-HA) was prepared from rooster comb HA (average molecular weight 400 kDa) as described with modifications^18^. Instead, we used TRITC, due to its superior photo stability. Fl-HA was used at a concentration of ≈170 pm. As an inert macromolecular tracer fluorescent, 500-kDa dextran (fl-Dextran) at ≈170 pm was used.

### Sample collection

The sample collection has been performed by the department of Orthopedic Surgery and Traumatology of the University Hospital in Bonn, Germany.

Samples have been taken of patients that underwent surgical procedures such as knee-Arthroscopy and Implantation of knee-Arthroplasty. The study was approved by the ethics committee of the University of Bonn, Germany, conducted in accordance with the approved guidelines and informed consent was obtained from the patients.

Patients who underwent knee-arthroscopy due to damage of the meniscus (medial and lateral were assigned to the appropriate group according to their joint status. After implanting the trocar of the arthroscope and before starting the lavage, samples of synovial fluid (maximum 5 ml) have been taken.

After inserting the arthroscope, the status of the cartilage has been analyzed. The patients have been structured in two different groups. If there was no severe cartilage damage (ICRS-score < 1) the patient was assigned to Group 1 (w/o-JD N = 5). If there have been severe cartilage damages (ICRS-score > 2) the patient has been assigned to Group 2 (w/-JD).

In patients that underwent implantation of a knee arthroplasty due to severe osteoarthritis, synovial fluid has been taken before opening joint capsula by using a cannula. These patients were grouped in Group 2(w/-JD total N = 16, N = 3 with ICRS-score > 2 and N = 13 with TKA)

Collected samples have been stored for a maximum of 48 h at 4 °C before single molecule measurement.

## Additional Information

**How to cite this article**: Kohlhof, H. *et al.* Single Molecule Microscopy Reveals an Increased Hyaluronan Diffusion Rate in Synovial Fluid from Knees Affected by Osteoarthritis. *Sci. Rep.* **6**, 21616; doi: 10.1038/srep21616 (2016).

## Supplementary Material

Supplementary Information

## Figures and Tables

**Figure 1:**
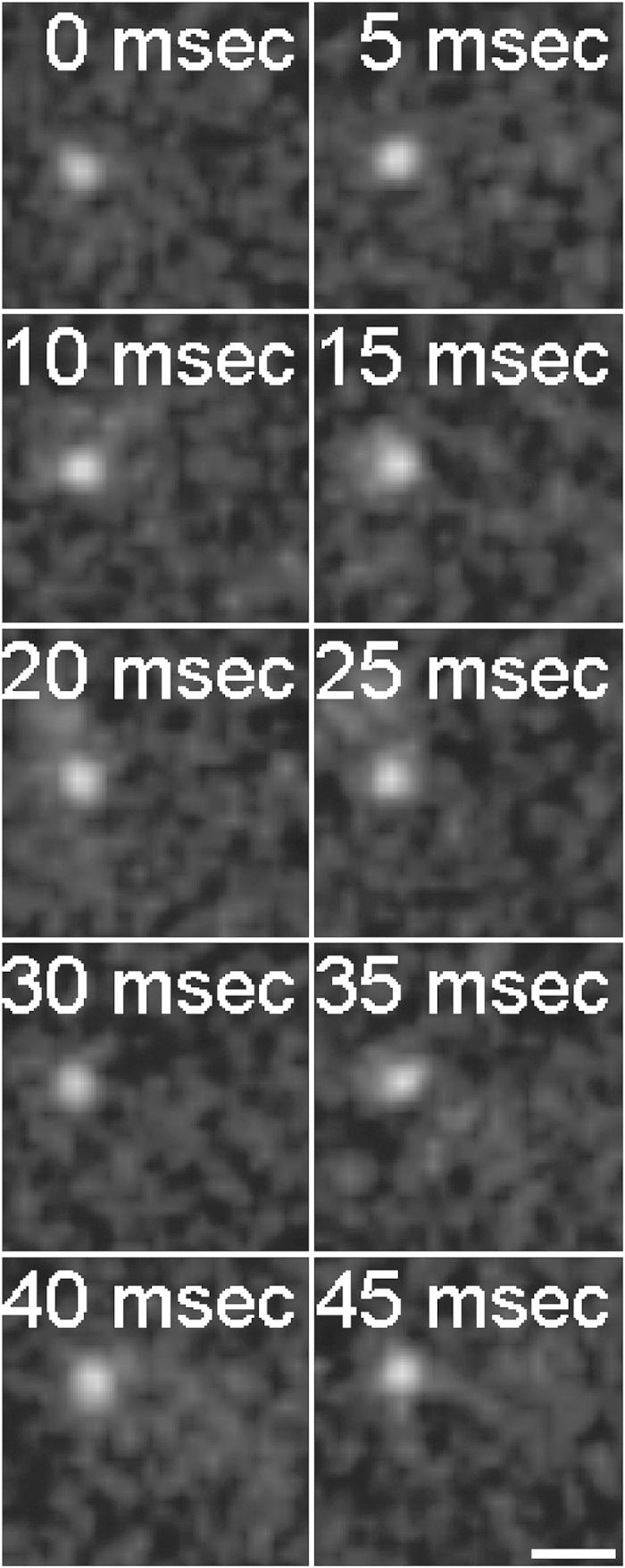
A single 400 kDa fl-Dextran molecule in synovial fluid imaged over 50 msec. The Brownian movements of the molecules are tracked. By analyzing, the distances the molecules move between single frames its diffusion coefficient is calculated. Bar 1 μm.
