# Supplementary material for: Single Molecule Microscopy Reveals an Increased Hyaluronan Diffusion Rate in Synovial Fluid from Knees Affected by Osteoarthritis
Source: Sci Rep. 2016 Feb 12;6:21616. doi: 10.1038/srep21616 (PMC4751503; doi:10.1038/srep21616)
Supplement: Supplementary Information [file srep21616-s1.pdf]

Supplementary information:

Single Molecule Microscopy Reveals an Increased Hyaluronan Diffusion Rate in Synovial Fluid from Knees Affected by Osteoarthritis

Kohlhof, Hendrik; Gravius, Sascha; Kohl, Sandro; Ahmad, Sufian S.; Randau, Thomas; Schmolders, Jan; Rommelspacher, Yorck; Friedrich, Max; Kaminski, Tim P.

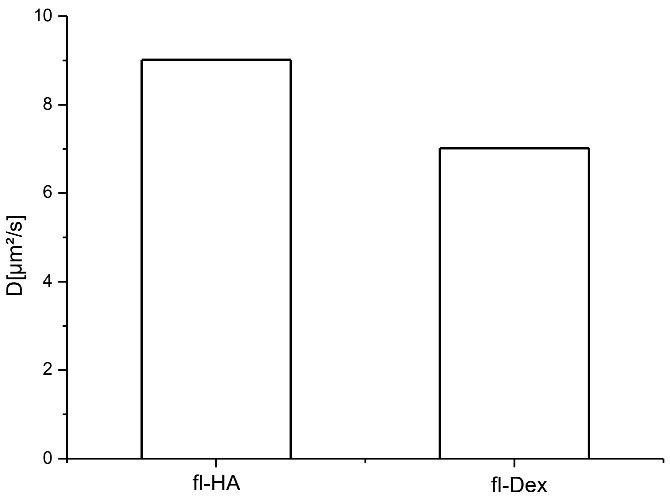

Supplementary Figure S1. Diffusion coefficients of fl-HA and fl-Dex in PBS-buffer at 22°C.
